# Unsupervised Word Embedding Learning by Incorporating Local and Global Contexts

**DOI:** 10.3389/fdata.2020.00009

**Published:** 2020-03-11

**Authors:** Yu Meng, Jiaxin Huang, Guangyuan Wang, Zihan Wang, Chao Zhang, Jiawei Han

**Affiliations:** ^1^Department of Computer Science, University of Illinois at Urbana-Champaign, Champaign, IL, United States; ^2^School of Computational Science and Engineering, College of Computing, Georgia Institute of Technology, Atlanta, GA, United States

**Keywords:** word embedding, unsupervised learning, word semantics, local contexts, global contexts

## Abstract

Word embedding has benefited a broad spectrum of text analysis tasks by learning distributed word representations to encode word semantics. Word representations are typically learned by modeling local contexts of words, assuming that words sharing similar surrounding words are semantically close. We argue that local contexts can only partially define word semantics in the unsupervised word embedding learning. Global contexts, referring to the broader semantic units, such as the document or paragraph where the word appears, can capture different aspects of word semantics and complement local contexts. We propose two simple yet effective unsupervised word embedding models that jointly model both local and global contexts to learn word representations. We provide theoretical interpretations of the proposed models to demonstrate how local and global contexts are jointly modeled, assuming a generative relationship between words and contexts. We conduct a thorough evaluation on a wide range of benchmark datasets. Our quantitative analysis and case study show that despite their simplicity, our two proposed models achieve superior performance on word similarity and text classification tasks.

## 1. Introduction

Unsupervised word representation learning, or word embedding, has shown remarkable effectiveness in various text analysis tasks, such as named entity recognition (Lample et al., 2016), text classification (Kim, 2014) and machine translation (Cho et al., 2014). Words and phrases, which are originally represented as one-hot vectors, are embedded into a continuous low-dimensional space. Typically, the mapping function is learned based on the assumption that words sharing similar local contexts are semantically close. For instance, the famous word2vec algorithm (Mikolov et al., 2013a,b) learns word representation from each word's local context window (i.e., surrounding words) so that local contextual similarity of words are preserved. The Skip-Gram architecture of word2vec uses the center word to predict its local context, and the CBOW architecture uses the local context to predict the center word. GloVe (Pennington et al., 2014) factorizes a global word-word co-occurrence matrix, but the co-occurrence is still defined upon local context windows.

**Figure 1:**
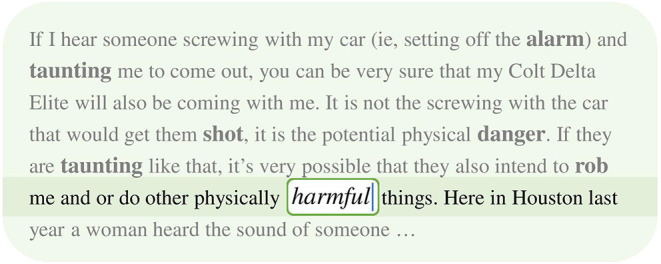
A text snippet from the 20 Newsgroup dataset. The transparent part represents the local context of the word “harmful.” The semitransparent part denotes the remainder of the document.

In this paper, we argue that apart from local context, another important type of word context—which we call *global context*—has been largely ignored by unsupervised word embedding models. Global context refers to the larger semantic unit that a word belongs to, such as a document or a paragraph. While local context reflects the local semantic and syntactic features of a word, global context encodes general semantic and topical properties of words in the document, which complements local context in embedding learning. Neither local context nor global context alone is sufficient for encoding the semantics of a word. For example, Figure 1 is a text snippet from the 20 Newsgroup dataset. When we only look at the local context window (the transparent part of Figure 1) of the word “harmful,” it is hard to predict if the center word should have positive or negative meaning. On the other hand, if we only know the entire document is about car robbery but do not have information about the local context, there is also no way to predict the center word. This example demonstrates that local and global contexts provide complementary information about the center word's semantics, and using either of them only may not be enough to capture the complete word semantics.

To the best of our knowledge, there is no previous study that *explicitly*^1^ models both local and global contexts to learn word representations. Topic models (Hofmann, 1999; Blei et al., 2003) essentially use global contexts to discover latent topics, by modeling documents as a mixture of latent topics and topics as distributions over words. In topic modeling, however, local contexts are completely ignored because word ordering information is discarded. Some studies along the embedding line learn word embeddings based on global contexts *implicitly*. HSMN (Huang et al., 2012), PTE (Tang et al., 2015), and Doc2Cube (Tao et al., 2018) take the average of word embedding in the document as the document representation and encourage similarity between word embedding and document embedding for co-occurred words and documents. However, these methods do not model global contexts explicitly because the document representations are essentially aggregated word representations and thus are not tailored for contextual representations. Moreover, both PTE and Doc2Cube require additional class information for text classification and thus are not unsupervised word embedding frameworks.

We propose two models that incorporate both local and global contexts for unsupervised word embedding learning. Our proposed models are surprisingly simple extensions of Skip-Gram and CBOW architectures of word2vec, by extending their objective functions to include a loss term corresponding to the global context. Despite our models' simplicity, we usea spherical generative model to show our models have theoretical bases: Under the assumption that there is a generative relationship between words and their contexts, our models essentially perform maximum likelihood estimation on the corpus with word representations as the parameters to be estimated.

Our contributions are summarized below:

1. We propose two unsupervised models that incorporate both local and global word contexts in word embedding learning, allowing them to provide complementary information for capturing word semantics.
2. We provide theoretical interpretations of the proposed models based on a spherical generative model, which shows equivalence between our models' objectives and maximum likelihood estimation on the corpus where word representations are parameters to be estimated.
3. We conduct a thorough evaluation on the word embedding quality trained on benchmark datasets. The two proposed models are superior to their word2vec counterparts and achieve superior performances on word similarity and text classification tasks. We also perform case studies to understand the properties of our models.

## 2. Related Work

In this section, we review related studies on word embedding, and categorize them into three classes according to the type of word context captured by the model.

### 2.1. Local Context Word Embedding

Most unsupervised word embedding frameworks learn word representations by preserving the local context similarity of words. The underlying assumption is that similar surrounding words imply similar semantics of center words. Distributed word representation is first proposed in Bengio et al. (2000) to maximize the conditional probability of the next word given the previous few words, which act as the local context. The definition of the local context is later extended in Collobert et al. (2011) to include not only the preceding words, but also the succeeding ones. Afterwards, the most famous word embedding framework, word2vec (Mikolov et al., 2013a,b), proposes two models that capture local context similarity. Specifically, word2vec's Skip-Gram model (Mikolov et al., 2013b) maximizes the probability of using the center word to predict its surrounding words; word2vec's CBOW model (Mikolov et al., 2013a), by symmetry, uses the local context to predict the center word. It is also shown in Levy and Goldberg (2014) that word2vec's Skip-Gram model with negative sampling is equivalent to factorizing a shifted PMI matrix. Another word embedding framework, GloVe (Pennington et al., 2014), learns embedding by factorizing a so-called global word-word co-occurrence matrix. However, the co-occurrence is still defined upon local context windows, so GloVe essentially captures local context similarity of words as well.

### 2.2. Global Context Word Embedding

There have been previous studies that incorporate global context, i.e., the document a word belongs to, into word embedding learning. Doc2Vec (Le and Mikolov, 2014) finds the representation for a paragraph or a document by training document embedding to predict words in the document. Although word embedding is trained simultaneously with the document embedding, the final goal of Doc2Vec is to obtain document embedding instead of word embedding, and documents are treated as the representation learning target but not as context for words.

A few recent papers incorporate global context implicitly into network structures where word embeddings are learned. PTE (Tang et al., 2015) and Doc2Cube (Tao et al., 2018) construct word-document network and encode word-document co-occurrence frequency in the edge weights to enforce embedding similarity between co-occurred words and documents. However, PTE and Doc2Cube do not explicitly model global context because document representations are simply the averaged word embedding. Another notable difference from unsupervised word embedding is that they also rely on another word-label network which requires class-related information to optimize the word embedding for text classification purposes. Hence, the embedding is trained under semi-supervised/weakly-supervised settings and does not generalize well to other tasks.

### 2.3. Joint Context Word Embedding

There have been a few attempts to incorporate both local and global contexts in word embedding. (Huang et al., 2012) proposes a neural language model which uses global context to disambiguate upon local context. Specifically, the framework conducts word sense discrimination for polysemy by learning multiple embeddings per word according to the document that the word token appears in. However, the document embedding is directly computed as the weighted average of word embeddings and is not tailored for contextual representation. In this paper, we explicitly learn document embedding as global context representation, so that local and global context representations clearly capture different aspects of word contexts. Topic word embeddings (Liu et al., 2015) and Collaborative Language Model (Xun et al., 2017) share the similar idea that topic modeling [e.g., LDA (Blei et al., 2003)] benefits word embedding learning by relating words with topical information. However, these types of framework suffer from the same major problems as topic modeling does: (1) They require prior knowledge about the number of latent topics in the corpus, which may not be always available under unsupervised settings; (2) Due to the local optimal solutions given by the topic modeling inference algorithm, the instability in topic discovery results in instability in word embedding as well. Our proposed models learn document embedding to represent global context and do not rely on topic modeling. The most relevant framework to our design is Spherical Text Embedding (Meng et al., 2019a) which jointly models word-word and word-paragraph co-occurrence statistics on the sphere.

## 3. Definitions and Preliminaries

In this section, we provide the meanings of the notations used in this paper in Table 1 and introduce the necessary preliminaries for understanding our design and interpretations of the proposed models.

**Table 1:** Notations and meanings.

**Notation**	**Meaning**
***u***_*w*_, ***v***_*w*_	The “input” and “output” vector representation of word *w*.
***d***	The vector representation of document *d*.
|*d*|	The length of document *d*.
$C_{L}\left(w,d\right),C_{G}\left(w,d\right)$	The local and global context of a word *w* ∈ *d*.
$D=\left\{d_{i}\right\}{}_{i=1}^{\left|D\right|}$	The text corpus represented by the set of documents.
$V=\left\{w_{i}\right\}{}_{i=1}^{\left|V\right|}$	The corpus vocabulary represented by the set of unique word tokens.
*h*	Local context window size.
*p*	Word and document vector dimension.
𝕌^*p*−1^	The unit sphere in ℝ^*p*^.

**Definition 1** (Local Context). We represent each document *d* as a sequence of words *d* = *w*_1_*w*_2_…*w*_*n*_. The local context $C_{L}\left(w_{i},d\right)$ of a word *w*_*i*_ ∈ *d* refers to all other words appearing in the local context window of *w*_*i*_ (i.e., *h* words before and after *w*_*i*_) in document *d*. Formally, $w_{j}\in C_{L}\left(w_{i},d\right)$ if *w*_*j*_ ∈ *d, i* − *h* ≤ *j* ≤ *i* + *h, i* ≠ *j*.

**Definition 2** (Global Context). The global context $C_{G}\left(w,d\right)$ of a word *w* with regard to *d* refers to the relationship that *w* appears in *d*. Formally, $C_{G}\left(w,d\right)=\left\{d\right\}$ if *w* ∈ *d*, and $C_{G}\left(w,d\right)=\emptyset$ otherwise.

**Definition 3** (The von Mises Fisher (vMF) distribution). A unit random vector ***x*** ∈ 𝕊^*p*−1^ ⊂ ℝ^*p*^ has the *p*-variate von Mises Fisher distribution *vMF*_*p*_(***μ***, κ) if its probability density function is

$$f(x;\;\mu ,\kappa )=c_{p}(\kappa )\text{ exp}(\kappa x^{⊤}\;\mu ),$$

where κ ≥ 0 is the concentration parameter, ||***μ***|| = 1 is the mean direction and the normalization constant *c*_*p*_(κ) is given by

$$c_{p}(\kappa )=\frac{\kappa^{p/2-1}}{{\left(2\pi \right)}^{p/2}I_{p/2-1}(\kappa )},$$

where *I*_*r*_(·) represents the modified Bessel function of the first kind at order *r*, as defined in Definition 4.

**Definition 4** (Modified Bessel Function of the First Kind). The modified Bessel function of the first kind of order *r* can be defined as (Mardia and Jupp, 2009):

$$I_{r}(\kappa )=\frac{{\left(\kappa /2\right)}^{r}}{\Gamma \left(r+\frac{1}{2}\right)\Gamma \left(\frac{1}{2}\right)}\int_{0}^{\pi }\text{ exp}(\kappa \text{ cos }\theta ){\left(\text{sin }\theta \right)}^{2r}d\theta ,$$

where $\Gamma \left(x\right)=\int_{0}^{\infty }\exp\left(-t\right)t^{x-1}dt$ is the gamma function.

## 4. Models

In this section, we introduce the two models built upon the word2vec framework that incorporate both global and local contexts in unsupervised word embedding learning.

### 4.1. Joint CBOW Model

The Joint CBOW model adopts the similar idea of word2vec's CBOW model (Mikolov et al., 2013a). Specifically, the model tries to predict the current word given its contexts. The objective has two components: the loss of using local context for prediction and the loss of using global context for prediction.

We define the loss of local context as below which encourages the model to correctly predict a word using its local context window:

(1) $$\begin{array}{l} {ℒ}_{local}=-\sum_{d\in D}\sum_{1\leq i\leq |d|}\text{log }p(w_{i}|C_{L}(w_{i},d))\\ \;=-\sum_{d\in D}\sum_{1\leq i\leq |d|}\text{log }p(w_{i}|w_{i-h},\ldots ,w_{i-1},w_{i+1},\ldots ,w_{i+h}). \end{array}$$

Following Mikolov et al. (2013a), we define the conditional probability to be

(2) $$p(w_{i}|w_{i-h},\ldots ,w_{i-1},w_{i+1},\ldots ,w_{i+h})=\frac{\text{ exp}({\bar{u}}_{w_{i}}^{⊤}v_{w_{i}})}{\sum_{w^{\prime }\in V}\text{ exp}({\bar{u}}_{w_{i}}^{⊤}v_{w^{\prime }})},$$

where ${\bar{u}}_{w_{i}}=\underset{-h\leq j\leq h,j\neq 0}{\sum }u_{w_{i+j}}/\|\underset{-h\leq j\leq h,j\neq 0}{\sum }u_{w_{i+j}}\|$ is the normalized sum of vector representations of words in the local context window of *w*_*i*_.

We define the loss of global context as below which encourages the model to correctly predict a word using the document it belongs to:

(3) $$\begin{array}{l} {ℒ}_{global}=-\sum_{d\in D}\sum_{1\leq i\leq |d|}\text{log }p(w_{i}|C_{G}(w_{i},d))\\ \;=-\sum_{d\in D}\sum_{1\leq i\leq |d|}\text{log }p(w_{i}|d). \end{array}$$

We define the conditional probability to be

(4) $$p(w_{i}|d)=\frac{\text{ exp}(v_{w_{i}}^{⊤}d)}{\sum_{w^{\prime }\in V}\text{ exp}(v_{w^{\prime }}^{⊤}d)}.$$

The final objective is the sum of local context loss and global context loss weighted by a hyperparameter λ.

(5) $${ℒ}_{total}={ℒ}_{local}+\lambda {ℒ}_{global}.$$

We note that when λ = 1, the model places equal emphasis on local and global contexts. When λ < 1, local context matters more and vice versa.

### 4.2. Joint Skip-Gram Model

The Joint Skip-Gram model mirrors the Joint CBOW model in that the inputs and outputs are swapped, i.e., now the model tries to predict the contexts given the current word. Again, the objective consists of a local context loss and a global context loss.

We define the loss of local context as below which encourages the model to correctly predict a word's local context window:

(6) $$\begin{array}{l} {ℒ}_{local}=-\sum_{d\in D}\sum_{1\leq i\leq |d|}\text{log }\;p(C_{L}(w_{i},d)|w_{i})\\ \;=-\sum_{d\in D}\sum_{1\leq i\leq |d|}\sum_{-h\leq j\leq h,j\neq 0}\text{log }p(w_{i+j}|w_{i}), \end{array}$$

Following (Mikolov et al., 2013b), we define the conditional probability to be

(7) $$p(w_{j}|w_{i})=\frac{\text{ exp}(u_{w_{i}}^{⊤}v_{w_{j}})}{\sum_{w^{\prime }\in V}\text{ exp}(u_{w_{i}}^{⊤}v_{w^{\prime }})},$$

We define the loss of global context as below which encourages the model to correctly predict the document a word belongs to:

(8) $$\begin{array}{l} {ℒ}_{global}=-\sum_{d\in D}\sum_{1\leq i\leq |d|}\text{log }\;p(C_{G}(w_{i},d)|w_{i})\\ \;=-\sum_{d\in D}\sum_{1\leq i\leq |d|}\text{log }\;p(d|w_{i}). \end{array}$$

We define the conditional probability to be

(9) $$p(d|w_{i})=\frac{\text{ exp}(u_{w_{i}}^{⊤}d)}{\sum_{d^{\prime }\in D}\text{ exp}(u_{w_{i}}^{⊤}d^{\prime })}.$$

The final objective is the sum of local context loss and global context loss weighted by a hyperparameter λ.

(10) $${ℒ}_{total}={ℒ}_{local}+\lambda {ℒ}_{global}.$$

We will study the effect of λ in the experiment section.

## 5. Interpreting the Models

**Figure 2:**
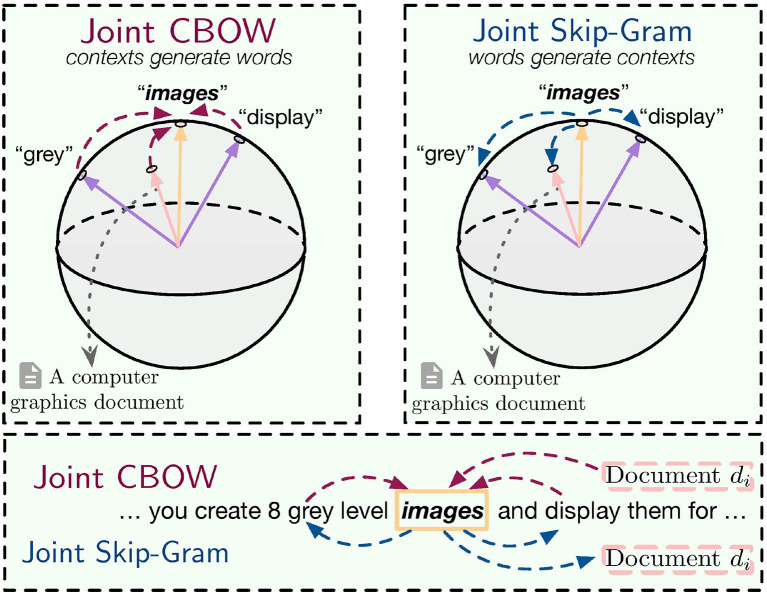
Joint CBOW and Joint Skip-Gram as generative models.

In this section, we propose a novel generative model to analyze the two models introduced in the previous section and show how they jointly incorporate global and local contexts. Overall, we assume there is *a generative relationship* between center words and contexts, i.e., either center words are generated from both local and global contexts (Joint CBOW), or local and global contexts are generated by center words (Joint Skip-Gram), as shown in Figure 2. A spherical distribution is used in the generative model where word vectors are treated as the parameters to be estimated.

### 5.1. The Spherical Generative Model

Before explaining Joint Skip-Gram and Joint CBOW, we first define the spherical distribution used in the generative model and show how it is connected with the conditional probability used in Joint Skip-Gram and Joint CBOW.

**Theorem 1**. When the corpus size and vocabulary size are infinite (i.e., |D|→∞ and |*V*| → ∞) and all word vectors and document vectors are assumed to be unit vectors^2^, generalizing the relationship of proportionality assumed in Equations (2), (4), (7), and (9), to the continuous cases results in the vMF distribution with the corresponding prior vector as the mean direction and constant 1 as the concentration parameter, i.e.,

$$\begin{array}{l} \underset{|V|\to \infty }{\lim}p(w_{i}|C_{L}(w_{i},d))=vMF_{p}({\bar{u}}_{w_{i}},1)=c_{p}(1)\text{ exp}(v_{w_{i}}^{⊤}{\bar{u}}_{w_{i}})\\ \;\underset{|V|\to \infty }{\lim}p(w_{i}|d)=vMF_{p}(d,1)=c_{p}(1)\text{ exp}(v_{w_{i}}^{⊤}d)\\ \;\underset{|V|\to \infty }{\lim}p(w_{j}|w_{i})=vMF_{p}(u_{w_{i}},\;1)=c_{p}(1)\text{ exp}(v_{w_{j}}^{⊤}u_{w_{i}})\\ \;\underset{|D|\to \infty }{\lim}p(d|w_{i})=vMF_{p}(u_{w_{i}},\;1)=c_{p}(1)\text{ exp}(d^{⊤}u_{w_{i}}) \end{array}$$

See Appendix for proof.

### 5.2. Joint CBOW as Words Generation

In this subsection, we show that Joint CBOW performs maximum likelihood estimation of the corpus assuming *local and global contexts generate words*. This assumption follows naturally how humans write articles: we first have a general idea about what the document is going to talk about, and then write down each word so that the word is coherent with both the meaning of the entire document (global context) and its surrounding words (local context).

We describe the details of the generative model below:

1. Underlying assumptions of local and global contexts.
  The global context representation ***d*** (equivalent to the document vector) encodes general semantic and topical information of the entire document and should be a constant vector; the local context representation ***l***_*i*_ encodes local semantic and syntactic information around *w*_*i*_ and should keep drifting slowly as the local context window shifts.
  Based on the above intuition, we assume ***d*** is fixed for each document *d*, while ***l***_*i*_ drifts slowly on the unit sphere in the embedding space with a small displacement between consecutive words. Finally, *w*_*i*_ is generated based on both ***d*** and ***l***_*i*_, i.e.,
  $$p(w_{i}\text{ gets generated})=p(w_{i}|l_{i})\cdot p(w_{i}|d).$$
2. Contexts generate words.
  Given the local context representation ***l***_*i*_ of *w*_*i*_ and global context representation ***d***, we assume the probability of a word being generated as *the center word* is given by the vMF distribution with the context representation as the mean direction and 1 as the concentration parameter:
  (11) $$p(w_{i}|l_{i})=vMF_{p}(l_{i},1)=c_{p}(1)\text{ exp}(v_{w_{i}}^{⊤}l_{i}),$$
  (12) $$p(w_{i}|d)=vMF_{p}(d,1)=c_{p}(1)\text{ exp}(v_{w_{i}}^{⊤}d).$$
  where we will derive the explicit representation of the local context representation ***l***_*i*_ later.
  Recall that in Joint CBOW, each word plays two roles: (1) center word and (2) context word for other words. Given the local context representation ***l***_*i*_ of *w*_*i*_, we assume the probability of a word being generated as *the context word* (we use *u*_*i*_ to denote the word is viewed as a context word instead of a center word) is also given by the vMF distribution:
  (13) $$p(u_{i}|l_{i})=vMF_{p}(l_{i},1)=c_{p}(1)\text{ exp}(u_{w_{i}}^{⊤}l_{i}),$$

Now we are ready to use the above generative model for explaining the relationship between Joint CBOW and text generation. We begin with deriving the explicit representation of ***l***_*i*_. Let U be the set of embedding of context words around word *w*_*i*_, i.e.,

$$U=\{u_{w_{i+j}}\in {𝕊}^{p-1}|u_{w_{i+j}}\text{ follows }vMF_{p}(l_{i},1),-h\leq j\leq h,j\neq 0\},$$

then we use the maximum likelihood estimates (see Appendix) to find $\hat{l_{i}}$:

$${\hat{l}}_{i}=\frac{\sum_{-h\leq j\leq h,j\neq 0}u_{w_{i+j}}}{\left\|\sum_{-h\leq j\leq h,j\neq 0}u_{w_{i+j}}\right\|}.$$

Now we view word vector representations ***v***_*w*_*i*__, ***u***_*w*_*i*+*j*__ and document representation ***d*** as parameters of the text generation model to be estimated, and write the likelihood of the corpus D as:

$$P(D|v_{w_{i}},\;u_{w_{i+j}},\;d)=\prod_{d\in D}\prod_{1\leq i\leq |d|}p(w_{i}|{\hat{l}}_{i})\;\cdot \;p(w_{i}|d).$$

When the corpus size is finite, we have to turn the equality in Equations (11) and (12) to proportionality, i.e., $p\left(w_{i}|l_{i}\right)\propto c_{p}\left(1\right)\exp\left(v_{w_{i}}^{⊤}l_{i}\right)$ and $p\left(w_{i}|d\right)\propto c_{p}\left(1\right)\exp\left(v_{w_{i}}^{⊤}d\right)$. Then the explicit expression of $p\left(w_{i}|\hat{l_{i}}\right)$ and that of *p*(*w*_*i*_∣***d***) become Equations (2) and (4), respectively.

The log-likelihood of the corpus D is:

$$\begin{array}{l} \text{log }P(D|v_{w_{i}},u_{w_{i+j}},d)\\ =\sum_{d\in D}\sum_{1\leq i\leq |d|}\left(\text{log }p(w_{i}|\hat{l_{i}})+\text{log }p(w_{i}|d)\right)\\ =\sum_{d\in D}\sum_{1\leq i\leq |d|}\left(\text{log }\frac{\text{ exp}(v_{w_{i}}^{⊤}{\hat{l}}_{i})}{\sum_{w^{\prime }}\text{ exp}(v_{w^{\prime }}^{⊤}{\hat{l}}_{i})}+\text{log }\frac{\text{ exp}(v_{w_{i}}^{⊤}d)}{\sum_{w^{\prime }}\text{ exp}(v_{w^{\prime }}^{⊤}d)}\right)\\ =-\left({ℒ}_{local}+{ℒ}_{global}\right), \end{array}$$

where $L_{local}$ and $L_{global}$ correspond to the local context loss (Equation 1) and the global context loss (Equation 3) of the Joint CBOW model, respectively. The only difference between the log-likelihood here and the Joint CBOW objective is that log-likelihood assumes equal weights on local and global contexts (λ = 1 in Equation 5).

Therefore, Joint CBOW performs maximum likelihood estimation on the text corpus with the assumption that words are generated by their contexts.

### 5.3. Joint Skip-Gram as Contexts Generation

In this subsection, we show that Joint Skip-Gram performs maximum likelihood estimation of the corpus assuming *center words generate their local and global contexts*, reversing the generation relationship assumed in Joint CBOW.

We describe the details of the generative model below:

1. Underlying assumptions of local and global contexts.
  The local context of a word *w*_*i*_ carries its local semantic and syntactic information and is assumed to be generated according to the semantics of *w*_*i*_. Further, we assume each context word in the local context window is generated independently, i.e.,
  $$p(C_{L}(w_{i},d)\text{ gets generated})=\prod_{w_{j}\in C_{L}(w_{i},d)}p(w_{j}|w_{i}).$$
  The global context of word *w*_*i*_ carries the global semantics of the entire document *d* that *w*_*i*_ belongs to and is assumed to be generated collectively by all the words in *d*, i.e.,
  $$p(C_{G}(w_{i},d)\text{ gets generated})=\prod_{w_{j}\in d}p(d|w_{j}).$$
2. Words generate contexts.
  Given the word representation ***u***_*w*_*i*__ of *w*_*i*_, we assume the local and global contexts of *w*_*i*_ are generated from the vMF distribution with ***u***_*w*_*i*__ as the mean direction and 1 as the concentration parameter:
  (14) $$p(w_{j}|w_{i})=vMF_{p}(u_{w_{i}},1)=c_{p}(1)\text{ exp}(v_{w_{i}}^{⊤}u_{w_{i}}),$$
  (15) $$p(d|w_{i})=vMF_{p}(u_{w_{i}},1)=c_{p}(1)\text{ exp}(d^{⊤}u_{w_{i}}).$$

Now we are ready to write out the likelihood of the collection of local and global contexts in the entire corpus $C=C_{L}\cup \;C_{G}$:

$$\begin{array}{l} P(C|u_{w_{i}},v_{w_{i+j}},d)=P(C_{L}|u_{w_{i}},v_{w_{i+j}},d)\cdot P(C_{G}|u_{w_{i}},v_{w_{i+j}},d)\\ \;=\left(\prod_{d\in D}\prod_{1\leq i\leq |d|}\prod_{w_{j}\in C_{L}(w_{i},d)}p(w_{j}|w_{i})\right)\\ \;\left(\prod_{d\in D}\prod_{1\leq i\leq |d|}p(d|w_{i})\right). \end{array}$$

When the corpus size is finite, we have to turn the equality in Equations (14) and (15) to proportionality, i.e., $p\left(w_{j}|w_{i}\right)\propto c_{p}\left(1\right)\exp\left(v_{w_{i}}^{⊤}u_{w_{i}}\right)$ and $p\left(d|w_{i}\right)\propto c_{p}\left(1\right)\exp\left(d^{⊤}u_{w_{i}}\right)$. Then the explicit expression of *p*(*w*_*j*_∣*w*_*i*_) and *p*(*d*∣*w*_*i*_) will become Equations (7) and (9), respectively.

The log-likelihood of the contexts C is:

$$\begin{array}{l} \text{log }P(C|u_{w_{i}},v_{w_{i+j}},d)=\sum_{d\in D}\sum_{1\leq i\leq |d|}\sum_{-h\leq j\leq h,j\neq 0}\text{log }p(w_{i+j}|w_{i})\\ \;+\sum_{d\in D}\sum_{1\leq i\leq |d|}\text{log }p(d|w_{i})\\ \;=\sum_{d\in D}\sum_{1\leq i\leq |d|}\sum_{-h\leq j\leq h,j\neq 0}\text{log }\frac{\text{exp}(u_{w_{i}}^{⊤}v_{w_{j}})}{\sum_{w^{\prime }\in V}\text{exp}(u_{w_{i}}^{⊤}v_{w^{\prime }})}\\ \;+\sum_{d\in D}\sum_{1\leq i\leq |d|}\text{log }\frac{\text{exp}(u_{w_{i}}^{⊤}d)}{\sum_{d^{\prime }\in D}\text{exp}(u_{w_{i}}^{⊤}d^{\prime })}\\ \;=-\left({ℒ}_{local}+{ℒ}_{global}\right), \end{array}$$

where $L_{local}$ and $L_{global}$ correspond to the local context loss (Equation 6) and the global context loss (Equation 8) of the Joint Skip-Gram model, respectively. The only difference between the log-likelihood here and the Joint Skip-Gram objective is that log-likelihood assumes equal weights on local and global contexts (λ = 1 in Equation 10).

Therefore, Joint Skip-Gram performs maximum likelihood estimation on the text corpus with the assumption that contexts are generated by words.

## 6. Experiments

In this section, we empirically evaluate the word embedding quality trained by our proposed models and conduct a set of case studies to understand the properties of our models.

### 6.1. Datasets

We use the following benchmark datasets for both word embedding training and text classification evaluation. The dataset statistics are summarized in Table 2.

**Table 2:** Dataset statistics.

**Dataset**	**# Train/# Test**	**# Classes**	**Avg. doc. length**
**20News**	11, 314/7, 532	20	396
**Reuters**	5, 485/2, 189	8	105

- **20News**: The 20 Newsgroup dataset^3^ contains newsgroup documents partitioned nearly evenly across 20 different newsgroups. We follow the same train/test split of the “bydate” version.
- **Reuters**: We use the 8-class version of the Reuters-21578 dataset^4^ following (Kusner et al., 2015; Xun et al., 2017) with the same train/test split as described in Sebastiani (2002).

### 6.2. Baselines and Ablations

We compare our models with the following baseline methods:

- **Skip-Gram** (Mikolov et al., 2013b) and **CBOW** (Mikolov et al., 2013a): The two models of the word2vec^5^ framework. **Skip-Gram** uses the center word to predict its local context, and **CBOW** uses local context to predict the center word.
- **GloVe** (Pennington et al., 2014): **GloVe**^6^ learns word embedding by factorizing a global word-word co-occurrence matrix where the co-occurrence is defined upon a fix-sized context window.
- **DM** and **DBOW** (Le and Mikolov, 2014): The two models of the Doc2Vec^7^ framework. **DM** uses the concatenation of word embeddings and document embedding to predict the next word, and **DBOW** uses the document embedding to predict the words in a window. Although Doc2Vec is originally used for learning paragraph/document representation, it also learns word embedding simultaneously. We evaluate the word embedding trained by Doc2Vec.
- **HSMN** (Huang et al., 2012): **HSMN**^8^ uses both local and global contexts to predict the next word in the sequence. The local context representation is obtained by concatenating the embedding of words preceding the next word, and the global context representation is simply the weighted average of all word embedding in the document.
- **PTE** (Tang et al., 2015): Predictive Text Embedding (**PTE**)^9^ constructs heterogeneous networks that encode word-word and word-document co-occurrences as well as class label information. It is originally trained under semi-supervised setting (i.e., labeled documents are required). We adapt it to unsupervised setting by pruning its word-label network.
- **TWE** (Liu et al., 2015): Topical word embedding (**TWE**)^10^ has three models for incorporating topical information into word embedding with the help of topic modeling. **TWE** requires prior knowledge about the number of latent topics in the corpus and we provide it with the correct number of classes of the corresponding corpus. We run all three models of **TWE** and report the best performance.

We compare our models with the following ablations:

- **Concat Skip-Gram** and **Concat CBOW**: The ablation of Joint Skip-Gram and Joint CBOW, respectively. We train Joint Skip-Gram and Joint CBOW twice with λ = 0 (only local context is captured) and λ = ∞ (only global context is captured). Then we concatenate the two embeddings so that the resulting embedding contains both local and global context information, but with two types of contexts trained independently. For fair comparison, the embedding dimension of λ = 0 and λ = ∞ cases is set to be $\frac{p}{2}$ so that the embedding dimension after concatenation is *p*, equal to that of Joint Skip-Gram and Joint CBOW.

### 6.3. Implementation Details and Settings

Because the full softmax in Equations (2), (4), (7), and (9) results in computational complexity proportional to the vocabulary size, we adopt the negative sampling strategy (Mikolov et al., 2013b) for efficient approximation.

We first pre-process the corpus by getting rid of infrequent words that appear < 5 times in the corpus. For fair comparison, we set the hyperparameters as below for all methods: word embedding dimension^11^ *p* = 100, local context window size *h* = 5, number of negative samples *k* = 5, number of training iterations on the corpus *iter* = 10. Other parameters (if any) are set to be the default values of the corresponding algorithm. Our method has an additional hyperparameter λ that balances between the importance of local and global contexts. We empirically find λ = 1.5 to be the optimal choice in general, so we report the performances of our models by setting λ = 1.5 for all tests.

### 6.4. Word Similarity Evaluation

In the first set of evaluation, we are interested in how well the word embedding captures similarity between word pairs. We use the following test datasets for evaluation: WordSim-353 (Finkelstein et al., 2001), MEN (Bruni et al., 2014), and SimLex-999 (Hill et al., 2015). These datasets contain word pairs with human-assigned similarity scores. We first train word embedding on **20News** dataset^12^, and then rank word pair similarity according to their cosine similarity value in the embedding space. Finally, we compare the ranking given by the word embedding with the ranking given by human ratings. We use both Spearman's rank correlation ρ and Kendall's rank correlation τ as measures with out-of-vocabulary word pairs excluded from the test sets.

The word similarity evaluation results are shown in Table 3. We observe that Joint Skip-Gram and Joint CBOW achieve the best performances under two metrics across three test sets. The fact that Joint Skip-Gram and Joint CBOW outperform **Skip-Gram**, **CBOW**, and **GloVe** demonstrates that by capturing global context in additional to local context, our model is able to rank word similarity more concordantly with human ratings. Comparing Joint Skip-Gram and Joint CBOW with **DM**, **DBOW**, and **PTE**, we show that our models are more effective in leveraging global context to capture word similarity. Our models also do better than **HSMN**, **TWE**, **Concat Skip-Gram**, and **Concat CBOW**, showing superiority in jointly incorporating local and global contexts.

**Table 3:** Word similarity evaluation.

**Methods**	**WordSim-353**		**Men**		**SimLex-999**	
	**ρ**	**τ**	**ρ**	**τ**	**ρ**	**τ**
**Skip-Gram**	0.430	0.293	0.303	0.206	0.153	0.104
**CBOW**	0.410	0.284	0.349	0.241	0.109	0.074
**GloVe**	0.207	0.140	0.196	0.134	0.042	0.028
**DBOW**	0.378	0.257	0.341	0.234	0.116	0.078
**DM**	0.367	0.254	0.305	0.209	0.116	0.079
**HSMN**	0.103	0.070	0.146	0.100	0.027	0.018
**PTE**	0.312	0.209	0.177	0.120	0.162	0.108
**TWE**	0.227	0.155	0.210	0.144	0.140	0.093
**Concat skip-gram**	0.369	0.248	0.324	0.221	0.163	0.111
**Concat CBOW**	0.413	0.283	0.350	0.240	0.110	0.073
Joint Skip-Gram	0.464	0.319	**0.375**	**0.256**	0.181	0.121
Joint CBOW	**0.473**	**0.326**	0.374	**0.256**	**0.192**	**0.131**

*Bold values denote the best performance among all methods*.

### 6.5. Text Classification Evaluation

In the second set of evaluation, we use a classical downstream task in NLP, text classification, to evaluate the quality of word embedding. For each of the two datasets described in section 6.1, we train a one-vs-rest logistic regression classifier on the training set and apply it on the testing set. The document features are obtained by averaging all word embedding vectors in the document, and the word embedding is trained on the training set of the corresponding dataset. We use Micro-F1 and Macro-F1 scores as metrics for classification performances, as in (Meng et al., 2018, 2019c).

The text classification performance is reported in Table 4. Under all cases, the best performance is achieved by either Joint Skip-Gram or Joint CBOW. Joint Skip-Gram and Joint CBOW give constantly better results than **Skip-Gram** and **CBOW**, respectively. This shows that global context enriches word embedding with topical semantics which is beneficial for the text classification task. Apart from the fact that our joint models achieve state-of-the-art performances as unsupervised word embedding for text classification, another interesting finding is that **Concat Skip-Gram** and **Concat CBOW** are pretty strong embedding baselines for text classification (outperforming **Skip-Gram** and **CBOW**), but are always inferior to Joint Skip-Gram and Joint CBOW. This indicates that the combination of local and global contexts indeed improves word embedding quality for classification tasks, but how to incorporate both types of contexts is also important—training jointly on local and global contexts is more effective than training independently on either context and then performing post-processing to obtain concatenated word embedding.

**Table 4:** Text classification evaluation.

**Methods**	**20News**		**Reuters**	
	**Macro-F1**	**Micro-F1**	**Macro-F1**	**Micro-F1**
**Skip-gram**	0.681	0.699	0.750	0.953
**CBOW**	0.653	0.668	0.866	0.965
**GloVe**	0.526	0.548	0.725	0.944
**DBOW**	0.687	0.703	0.796	0.950
**DM**	0.594	0.610	0.837	0.955
**HSMN**	0.385	0.431	0.200	0.736
**PTE**	0.700	0.718	0.776	0.957
**TWE**	0.608	0.632	0.616	0.916
**Concat Skip-Gram**	0.759	0.772	0.764	0.958
**Concat CBOW**	0.680	0.695	0.873	0.961
Joint Skip-Gram	**0.773**	**0.785**	0.854	0.962
Joint CBOW	0.736	0.753	**0.885**	**0.966**

*Bold values denote the best performance among all methods*.

### 6.6. Parameter Study

**Figure 3:**
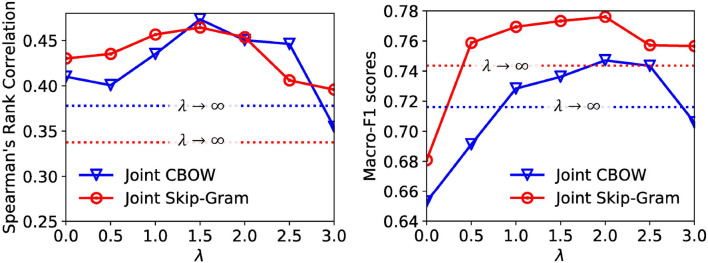
Hyperparameter study on word similarity **(left)** and text classification **(right)**.

In the previous subsections, we fix λ for both Joint Skip-Gram and Joint CBOW models for all evaluation tasks. In this subsection, we would like to explore the trade-off between local and global contexts in embedding learning. Specifically, we vary λ in the Joint Skip-Gram and Joint CBOW model with a 0.5 interval in range [0, 3] and ∞ (the performances of λ = ∞ are represented as horizontal dotted lines), and conduct word similarity evaluation on the WordSim-353 dataset and text classification evaluation on the **20News** dataset. The performances under different λ's for both models are shown in Figure 3. We observe that the optimal settings of both models for word similarity and text classification are λ = 1.5 and λ = 2.0, respectively. This verifies our arguments that combining both types of contexts achieves the best performances.

### 6.7. Running Time Study

We report the training time on **20News** dataset per iteration of all baselines in Table 5 to compare the training efficiency. All the models are run on a machine with 20 cores of Intel(R) Xeon(R) CPU E5-2680 v2 @ 2.80 GHz. Joint Skip-Gram and Joint CBOW have similar training time with their original counterparts and are more efficient than the other baselines, demonstrating their high efficiency.

**Table 5:** Running time evaluation on **20News** dataset.

**Methods**	**Skip-Gram**	**CBOW**	**GloVe**	**DBOW**	**DM**	**HSMN**	**PTE**	**TWE**	**JSG**	**JCBOW**
Running time (s)	29.8	24.7	31.2	41.9	35.7	44.6	48.8	>1,000	30.1	25.4

### 6.8. Case Studies

In this subsection, we perform a set of case studies to understand the properties of our models and why incorporating both local and global contexts leads to better word embedding. We conduct all the case studies on the **20News** dataset unless stated otherwise.

#### 6.8.1. Effect of Global Context

We are interested in why and how global context can be beneficial for capturing more complete word semantics. We set λ = ∞ and λ = 0 in Equation (10) so that the embedding trained by Joint Skip-Gram only captures the global/local context of words. We select a set of acronyms (e.g., *CMU* stands for Carnegie Mellon University.) and use their embedding to retrieve a few most similar words (measured by cosine similarity in the embedding space). In Table 6, we list five university acronyms and show the top words retrieved by the embedding trained with only global context and only local context, respectively. We observe that local context embedding retrieves nothing meaningful related to the acronyms, but global context embedding successfully finds the original word components of the acronyms. The reason is that each original word component usually does not share similar local context with the acronym (e.g., *CMU* and the single word “Carnegie” obviously have different surrounding words) despite their semantic similarity. However, the original word components and acronyms usually appear in same/similar documents, resulting in higher global context similarity. The insights gained from this case study can be generalized to other cases where words are semantically similar but syntactically dissimilar. Global context is effective in discovering semantic and topical similarity of words without enforcing syntactic similarity.

**Table 6:** Effect of global context on interpreting acronyms.

**Acronyms**	**Global (**λ = ∞**)**	**Local (**λ = 0**)**
CMU	**mellon**, **carnegie**, andrew, pa, pittsburgh	andrew, kfnjyea00uh, am2x, mr47, devineni
UIUC	**urbana**, **illinois**, uxa, **univ**, uchicago	uxa, ux4, ux1, mrcnext, cka52397
UNC	**chapel**, **carolina**, astro, images, usc	launchpad, gibbs, umr, lambada, jge
Caltech	**california**, gap, **institute**, keith, **technology**	juliet, jafoust, lmh, henling, bdunn
JHU	**johns**, camp, **hopkins**, nation, grand	pablo, hasch, iglesias, davidk, atlantis

*Bold values denote the best performance among all methods*.

#### 6.8.2. Different Contexts Capture Different Aspects of Word Similarity

Word similarity has different aspects. Words can be semantically similar but syntactically dissimilar and vice versa. For example, antonyms have opposite semantics (e.g., *good* vs. *bad*) but are syntactically similar and may occur with similar short surrounding contexts. We list a set of antonyms and provide their embedding cosine similarity when different types of context are captured by Joint Skip-Gram (Global, λ = ∞; Local, λ = 0; Joint, λ = 1.5) as shown in Table 7.

**Table 7:** Cosine similarity of antonym embeddings trained with different contexts.

**Antonyms**	**Global (**λ = ∞**)**	**Local (**λ = 0**)**
Good—bad	0.3150	0.7127
Happy—unhappy	0.3911	0.6178
Large—small	0.4871	0.7265
Increase—decrease	0.2663	0.7308
Enter—exit	0.2756	0.5553
Save—spend	−0.0388	0.4792

It can be observed that all antonyms have high cosine similarity when only local context is captured in embedding (λ = 0). On the other hand, antonym embeddings trained on global context (λ = ∞) have relatively low cosine similarity. The results verify our intuition that local context focuses more on syntactic similarity while global context emphasizes more on semantic or topical similarity of words. Our joint model strikes a balance between local and global contexts, and thus reflects both syntactic and semantic aspects of word similarity.

#### 6.8.3. Global Context Embedding Quality

**Figure 4:**
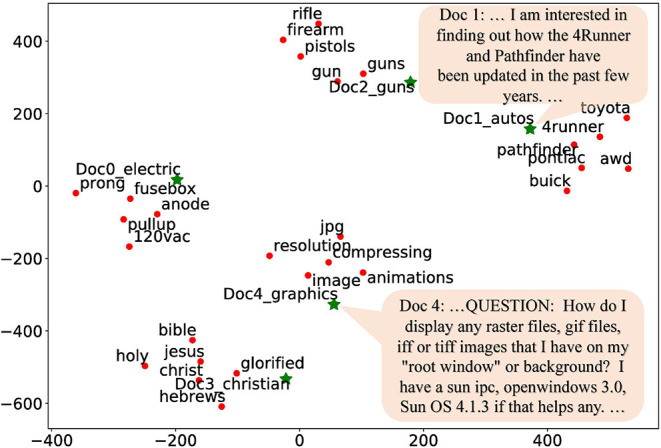
Word and document embedding visualization.

In the third set of case studies, we qualitatively evaluate the global context embedding by visualizing the document vectors together with word embeddings. We select five documents from five different topics of the **20News** dataset, and then select several topical related words for each document. The five topics are: *electric, automobiles, guns, christian*, and *graphics*. We apply t-SNE (van der Maaten and Hinton, 2008) to visualize both the document embedding and the word embedding in Figure 4, where green stars represent document embeddings and red dots represent word embeddings. Documents are indeed embedded close to their topical related words, implying that global context embeddings appropriately encode topical semantic information, which consequently benefits word embedding learning.

#### 6.8.4. Weakly-Supervised Text Classification

In the previous case study, we have shown word embedding and document embedding can be jointly trained unsupervisedly. It then becomes natural to consider the possibility to perform text classification without labeled documents. When only weak supervisions, such as class surface names (e.g., *politics, sports*) are available, the unsupervised word embedding quality becomes essential for text classification because there is no additional labeling for fine-tuning word embedding. WeSTClass (Meng et al., 2018, 2019c) models class semantics as vMF distributions in the word embedding space and applies a pretrain-refine neural approach to perform text classification under weak supervision. Doc2Cube (Tao et al., 2018) leverages word-document co-occurrences to embed class labels, words and documents in the same space and perform classification by comparing embedding similarity. We adopt the two frameworks and replace the original embedding with the embedding trained by our Joint Skip-Gram and Joint CBOW models. We perform weakly-supervised text classification on the training set of **Reuters** with class names as weak supervision and report the Macro-F1 and Micro-F1 scores in Table 8.

**Table 8:** Weakly-supervised text classification on Reuters.

**Methods**	**Macro-F1**	**Micro-F1**
WeSTClass	0.554	0.593
Doc2Cube	0.435	0.446
Doc2Cube w/Joint Skip-Gram	0.585	0.717
Doc2Cube w/Joint CBOW	0.570	0.700
WeSTClass w/Joint Skip-Gram	**0.717**	**0.801**
WeSTClass w/Joint CBOW	0.691	0.698

*Bold values denote the best performance among all methods*.

We show that we can achieve reasonably good text classification performances even without labeled documents, by fully leveraging the context information to capture more complete semantics in word embedding.

## 7. Discussions

In this section, we discuss several open issues and interesting directions for further exploration.

- How to choose appropriate global contexts in practice?
  In Definition 2, we defined global context to be the document in which a word appears. In practice, however, the global context of a word can flexibly refer to its belonging paragraph, or several sentences surrounding it, based on different application scenarios. For example, for short documents like a piece of review text, it is appropriate to use the entire document as the global context of a word. In long news articles or research papers, it might be more suitable to define the global context as the paragraph or the subsection a word appears in. Therefore, we recommend practitioners to experiment with different global context settings for different texts.
- Global context for other embedding training settings.
  In this work, we showed that using global contexts in addition to local contexts improves unsupervised word embedding quality since the two types of contexts capture complementary information about a word. Based on this observation, we may consider incorporating global contexts into other embedding learning settings. For example, in CatE (Meng et al., 2020) we improve the discriminative power of the embedding model over a specific set of user-provided categories with the help of global contexts, based on which a topic mining framework (Meng et al., 2019b) is further developed. We believe that there are many other tasks where global contexts can complement local contexts in training and fine-tuning embeddings.
- Embedding learning in the spherical space.
  It has been shown that directional similarity is more effective than Euclidean measurement in word similarity and clustering. Therefore, it might be beneficial to model both local and global contexts in the spherical space to train text embeddings of even better quality, like in JoSE (Meng et al., 2019a). Further exploration might involve using Riemannian optimization on the unit sphere or enforcing vector norm constraints to fine-tune text embeddings in downstream tasks.

## 8. Conclusions and Future Work

We propose two simple yet effective unsupervised word embedding learning models to jointly capture the complementary word contexts. Local context focuses more on the syntactic and local semantic aspect whereas global context provides information more regarding the general and topical semantics of words. Experiments show that incorporating both types of contexts achieves state-of-the-art performance on word similarity and text classification tasks. We provide a novel generative perspective to theoretically interpret the two proposed models. The interpretation might pave the path for several future directions:

- The global context may not be always defined as the document that a word appears in, because the generative relationship between a word and its corresponding sentence/paragraph might be stronger than that between a word and the entire document.
- Our current models (and the original word2vec framework) assume that the vMF distribution for generating words/contexts has constant 1 as the concentration parameter κ. However, the most appropriate κ might depend on vocabulary size, average document length in the corpus, etc. and can vary across different datasets. It will be interesting to explore how to set appropriate κ for even better word embedding quality.

## Data Availability Statement

Publicly available datasets were analyzed in this study. This data can be found here: http://qwone.com/~jason/20Newsgroups/, http://www.daviddlewis.com/resources/testcollections/reuters21578/.

## Author Contributions

YM and JHu contributed to the design of the models. YM, JHu, GW, and ZW implemented the models and conducted the experiments. YM, JHu, CZ, and JHa wrote the manuscript. All authors contributed to the manuscript revision, read, and approved the submitted version.

### Conflict of Interest

The authors declare that the research was conducted in the absence of any commercial or financial relationships that could be construed as a potential conflict of interest.

## Footnotes

^1^“Explicitly” means local context and global context have explicit and independent vector representations.

^2^This is similar to the constraint introduced in Meng et al. (2019a).

^3^http://qwone.com/~jason/20Newsgroups/

^4^http://www.daviddlewis.com/resources/testcollections/reuters21578/

^5^https://code.google.com/archive/p/word2vec/

^6^https://nlp.stanford.edu/projects/glove/

^7^https://radimrehurek.com/gensim/models/doc2vec.html

^8^http://ai.stanford.edu/~ehhuang/

^9^https://github.com/mnqu/PTE

^10^https://github.com/largelymfs/topical_word_embeddings

^11^Since the datasets used in our experiments are relatively small-scale, using higher embedding dimensions (e.g., *p* = 200, 300) does not lead to noticeably different results, so we only report the results with *p* = 100.

^12^In this work, we are interested in embedding quality when embeddings are trained on the local corpus where downstream tasks are carried out. In Meng et al. (2019a), we report the word similarity evaluation of embeddings trained on the Wikipedia dump.

## References

[B1] Bengio Y. Ducharme R. Vincent P. (2000). “A neural probabilistic language model,” in Conference on Neural Information Processing Systems (Denver, CO).

[B2] Blei D. M. Ng A. Y. Jordan M. I. (2003). Latent dirichlet allocation. J. Mach. Learn. Res. 3, 993–1022. 10.5555/944919.944937

[B3] Bruni E. Tran N.-K. Baroni M. (2014). Multimodal distributional semantics. J. Artif. Intell. Res. 49, 1–47. 10.1613/jair.4135

[B4] Cho K. van Merrienboer B. Gülçehre Ç. Bahdanau D. Bougares F. Schwenk H.. (2014). “Learning phrase representations using rnn encoder-decoder for statistical machine translation,” in Conference on Empirical Methods in Natural Language Processing (Doha).

[B5] Collobert R. Weston J. Bottou L. Karlen M. Kavukcuoglu K. Kuksa P. P. (2011). Natural language processing (almost) from scratch. J. Mach. Learn. Res. 12, 2493–2537. 10.5555/1953048.2078186

[B6] Finkelstein L. Gabrilovich E. Matias Y. Rivlin E. Solan Z. Wolfman G.. (2001). “Placing search in context: the concept revisited,” in WWW'01: Proceedings of the 10th International Conference on World Wide Web (Hong Kong).

[B7] Hill F. Reichart R. Korhonen A. (2015). Simlex-999: evaluating semantic models with (genuine) similarity estimation. Comput. Linguist. 41, 665–695. 10.1162/COLI_a_00237

[B8] Hofmann T. (1999). “Probabilistic latent semantic indexing,” in SIGIR'99: Proceedings of the 22nd Annual International ACM SIGIR Conference on Research and Development in Information Retrieval (Berkeley, CA).

[B9] Huang E. H. Socher R. Manning C. D. Ng A. Y. (2012). “Improving word representations via global context and multiple word prototypes,” in Proceedings of the 50th Annual Meeting of the Association for Computational Linguistics (Jeju Island).

[B10] Kim Y. (2014). “Convolutional neural networks for sentence classification,” in Proceedings of the 2014 Conference on Empirical Methods in Natural Language Processing (Doha). 10.18653/v1/d16-1076 PMC5300751 28191551

[B11] Kusner M. J. Sun Y. Kolkin N. I. Weinberger K. Q. (2015). “From word embeddings to document distances,” in Proceedings of the 32nd International Conference on Machine Learning.

[B12] Lample G. Ballesteros M. Subramanian S. Kawakami K. Dyer C. (2016). “Neural architectures for named entity recognition,” in Proceedings of the 2016 Conference of the North American Chapter of the Association for Computational Linguistics: Human Language Technologies (San Diego, CA).

[B13] Le Q. V. Mikolov T. (2014). “Distributed representations of sentences and documents,” in Proceedings of the 31st International Conference on Machine Learning (Beijing).

[B14] Levy O. Goldberg Y. (2014). “Neural word embedding as implicit matrix factorization,” in NIPS'14: Proceedings of the 27th International Conference on Neural Information Processing Systems (Montreal, QC).

[B15] Liu Y. Liu Z. Chua T.-S. Sun M. (2015). “Topical word embeddings,” in AAAI'15: Proceedings of the Twenty-Ninth AAAI Conference on Artificial Intelligence (Austin, TX).

[B16] Mardia K. V. Jupp P. E. (2009). Directional Statistics, Vol. 494. John Wiley & Sons.

[B17] Meng Y. Huang J. Wang G. Wang Z. Zhang C. Zhang Y.. (2020). “Discriminative topic mining via category-name guided text embedding,” in Proceedings of The Web Conference 2020 (WWW20) (Taipei).

[B18] Meng Y. Huang J. Wang G. Zhang C. Zhuang H. Kaplan L.. (2019a). “Spherical text embedding,” in 33rd Conference on Neural Information Processing Systems (NeurIPS 2019) (Vancouver, BC).

[B19] Meng Y. Huang J. Wang Z. Fan C. Wang G. Zhang C.. (2019b). “Topicmine: user-guided topic mining by category-oriented embedding,” in ACM SIGKDD Conference on Knowledge Discovery and Data Mining (KDD) (Anchorage, AK).

[B20] Meng Y. Shen J. Zhang C. Han J. (2018). “Weakly-supervised neural text classification,” in ACM International Conference on Information and Knowledge Management (CIKM) (Torino).

[B21] Meng Y. Shen J. Zhang C. Han J. (2019c). “Weakly-supervised hierarchical text classification,” in AAAI Conference on Artificial Intelligence (AAAI) (Honolulu, HI).

[B22] Mikolov T. Chen K. Corrado G. S. Dean J. (2013a). Efficient estimation of word representations in vector space. CoRR abs/1301.3781.

[B23] Mikolov T. Sutskever I. Chen K. Corrado G. S. Dean J. (2013b). “Distributed representations of words and phrases and their compositionality,” in NIPS'13: Proceedings of the 26th International Conference on Neural Information Processing Systems (Lake Tahoe, NV).

[B24] Pennington J. Socher R. Manning C. D. (2014). “Glove: global vectors for word representation,” in Proceedings of the 2014 Conference on Empirical Methods in Natural Language Processing (EMNLP) (Doha).

[B25] Sebastiani F. (2002). Machine learning in automated text categorization. ACM Comput. Surv. 34, 1–47. 10.1145/505282.505283

[B26] Tang J. Qu M. Mei Q. (2015). “Pte: predictive text embedding through large-scale heterogeneous text networks,” in KDD '15: Proceedings of the 21th ACM SIGKDD International Conference on Knowledge Discovery and Data Mining (Sydney, NSW).

[B27] Tao F. Zhang C. Chen X. Jiang M. Hanratty T. Kaplan L. M.. (2018). “Doc2cube: allocating documents to text cube without labeled data,” in 2018 IEEE International Conference on Data Mining (ICDM) (Singapore).

[B28] van der Maaten L. Hinton G. E. (2008). Visualizing data using t-SNE. J. Mach. Learn. Res. 9, 2579–2605.

[B29] Xun G. Li Y. Gao J. Zhang A. (2017). “Collaboratively improving topic discovery and word embeddings by coordinating global and local contexts,” in 23rd ACM SIGKDD Conference on Knowledge Discovery and Data Mining (Halifax, NS).

