# Supplementary material for: Unsupervised Word Embedding Learning by Incorporating Local and Global Contexts
Source: Front Big Data. 2020 Mar 11;3:9. doi: 10.3389/fdata.2020.00009 (PMC7931948; doi:10.3389/fdata.2020.00009)
Supplement: Supplementary file 1 [file Presentation_3.pdf]

APPENDIX

Lemma 1. The definite integral of power of $\sin$ on the interval $[0, \pi]$ is given by

$$J_p = \int_0^\pi (\sin x)^p dx = \frac{\sqrt{\pi}\Gamma\left(\frac{1+p}{2}\right)}{\Gamma\left(1+\frac{p}{2}\right)} \quad (p \in \mathbb{Z}^+),$$

where $\Gamma(x) = \int_0^\infty \exp(-t)t^{x-1}dt$ is the gamma function.

PROOF.

$$\begin{aligned} J_p &= \int_0^\pi (\sin x)^p dx \\ &= \left(-(\sin x)^{p-1}\cos x\right)\Big|_0^\pi + (p-1)\int_0^\pi (\sin x)^{p-2}(\cos x)^2 dx \\ &= 0 + (p-1)\int_0^\pi (\sin x)^{p-2}dx - (p-1)\int_0^\pi (\sin x)^p dx \\ &= (p-1)J_{p-2} - (p-1)J_p \end{aligned}$$

Therefore, $J_p = \frac{p-1}{p}J_{p-2}$.

Using the above iteration relationship and the property of gamma function $\Gamma(x+1) = x\Gamma(x)$, we write J_p using gamma function:

- When p is an even integer:

$$\begin{aligned} J_p &= \frac{p-1}{p}\frac{p-3}{p-2}\cdots\frac{1}{2}J_0 \\ &= \frac{(p-1)/2}{p/2}\frac{(p-3)/2}{(p-2)/2}\cdots\frac{1/2}{2/2}J_0 \\ &= \frac{\Gamma\left(\frac{1+p}{2}\right)/\Gamma\left(\frac{1}{2}\right)}{\Gamma\left(\frac{p}{2}+1\right)/\Gamma(1)}J_0 \end{aligned}$$

Plugging in the base case $J_0 = \pi$ and $\Gamma\left(\frac{1}{2}\right) = \sqrt{\pi}$, $\Gamma(1) = 1$, we prove that

$$J_p = \frac{\sqrt{\pi}\Gamma\left(\frac{1+p}{2}\right)}{\Gamma\left(1+\frac{p}{2}\right)} \quad (p \in \mathbb{Z}^+, p \text{ is even})$$

- When p is an odd integer:

$$\begin{aligned} J_p &= \frac{p-1}{p}\frac{p-3}{p-2}\cdots\frac{2}{3}J_1 \\ &= \frac{(p-1)/2}{p/2}\frac{(p-3)/2}{(p-2)/2}\cdots\frac{2/2}{3/2}J_1 \\ &= \frac{\Gamma\left(\frac{1+p}{2}\right)/\Gamma(1)}{\Gamma\left(\frac{p}{2}+1\right)/\Gamma\left(\frac{3}{2}\right)}J_1 \end{aligned}$$

Plugging in the base case $J_1 = 2$ and $\Gamma\left(\frac{3}{2}\right) = \frac{\sqrt{\pi}}{2}$, $\Gamma(1) = 1$, we prove that

$$J_p = \frac{\sqrt{\pi}\Gamma\left(\frac{1+p}{2}\right)}{\Gamma\left(1+\frac{p}{2}\right)} \quad (p \in \mathbb{Z}^+, p \text{ is odd})$$

Theorem 1. When the corpus size and vocabulary size are infinite (*i.e.*, $|\mathcal{D}| \to \infty$ and $|V| \to \infty$) and all word vectors and document vectors are assumed to be unit vectors, generalizing the relationship of proportionality assumed in Equations (2), (4), (7) and (9), to the continuous cases results in the vMF distribution with the corresponding prior vector as the mean direction and constant 1 as the concentration parameter, *i.e.*,

$$\lim_{|V|\to\infty} p(w_i \mid \mathcal{C}_L(w_i, d)) = vMF_p(\overline{\boldsymbol{u}}_{w_i}, 1) = c_p(1)\exp(\boldsymbol{v}_{w_i}^\top \overline{\boldsymbol{u}}_{w_i}) \tag{16}$$

$$\lim_{|V|\to\infty} p(w_i \mid d) = vMF_p(\boldsymbol{d}, 1) = c_p(1)\exp(\boldsymbol{v}_{w_i}^\top \boldsymbol{d}) \tag{17}$$

$$\lim_{|V|\to\infty} p(w_j \mid w_i) = vMF_p(\boldsymbol{u}_{w_i}, 1) = c_p(1)\exp(\boldsymbol{v}_{w_j}^\top \boldsymbol{u}_{w_i}) \tag{18}$$

$$\lim_{|\mathcal{D}|\to\infty} p(d \mid w_i) = vMF_p(\boldsymbol{u}_{w_i}, 1) = c_p(1)\exp(\boldsymbol{d}^\top \boldsymbol{u}_{w_i}) \tag{19}$$

PROOF. We give the proof for Equation (18). The proof for Equations (16), (17) and (19) can be derived similarly.

We generalize the relationship proportionality $p(w_j \mid w_i) \propto \exp(\boldsymbol{u}_{w_i}^\top \boldsymbol{v}_{w_j})$ in Equation (7) to the continuous case and obtain the following probability dense distribution:

$$\lim_{|V|\to\infty} p(w_j \mid w_i) = \frac{\exp(\boldsymbol{u}_{w_i}^\top \boldsymbol{v}_{w_j})}{\int_{\mathbb{S}^{p-1}} \exp(\boldsymbol{u}_{w_i}^\top \boldsymbol{v}_{w'}) d\boldsymbol{v}_{w'}} \triangleq \frac{\exp(\boldsymbol{u}_{w_i}^\top \boldsymbol{v}_{w_j})}{Z}, \tag{20}$$

where we denote the integral in the denominator as Z, and our goal becomes to prove the following equality[1]

$$Z = \frac{1}{c_p(1)}.$$

To evaluate the integral Z, we make the transformation to polar coordinates. Let $\boldsymbol{t} = Q\boldsymbol{v}_{w'}$, where $Q \in \mathbb{R}^{p\times p}$ is an orthogonal transformation so that $d\boldsymbol{t} = d\boldsymbol{v}_{w'}$. Moreover, let the first row of Q be $\boldsymbol{u}_{w_i}$ so

[1] An easy way to see this holds true (without formal proof) is to use the fact that the probability density function of vMF distribution integrates to 1 over the entire sphere.

that $t_1 = \boldsymbol{u}_{w_i}^\top \boldsymbol{v}_{w'}$. Then we use $(r, \theta_1, \ldots, \theta_{p-1})$ to represent the polar coordinates of $\boldsymbol{t}$ where $r = 1$ and $\cos\theta_1 = \boldsymbol{u}_{w_i}^\top \boldsymbol{v}_{w'}$. The transformation from Euclidean coordinates to polar coordinates is given by (Sra, 2007) via computing the determinant of the Jacobian matrix for the coordinate transformation:

$$d\boldsymbol{t} = r^{p-1} \prod_{j=2}^{p} (\sin\theta_{j-1})^{p-j} d\theta_{j-1}.$$

Then

$$Z = \int_0^\pi \exp(\cos\theta_1)(\sin\theta_1)^{p-2} d\theta_1 \prod_{j=3}^{p-1} \int_0^\pi (\sin\theta_{j-1})^{p-j} d\theta_{j-1} \int_0^{2\pi} d\theta_{j-1}.$$

By Lemma 1, we have

$$\prod_{j=3}^{p-1} \int_0^\pi (\sin\theta_{j-1})^{p-j} d\theta_{j-1} = \pi^{\frac{p-3}{2}} \frac{\Gamma\left(\frac{p-2}{2}\right)\Gamma\left(\frac{p-3}{2}\right)\cdots\Gamma(1)}{\Gamma\left(\frac{p-1}{2}\right)\Gamma\left(\frac{p-2}{2}\right)\cdots\Gamma\left(\frac{3}{2}\right)} = \frac{\pi^{\frac{p-3}{2}}}{\Gamma\left(\frac{p-1}{2}\right)}.$$

Then

$$\begin{aligned} Z &= \int_0^\pi \exp(\cos\theta_1)(\sin\theta_1)^{p-2} d\theta_1 \cdot \frac{\pi^{\frac{p-3}{2}}}{\Gamma\left(\frac{p-1}{2}\right)} \cdot 2\pi \\ &= \frac{2\pi^{\frac{p-1}{2}}}{\Gamma\left(\frac{p-1}{2}\right)} \int_0^\pi \exp(\cos\theta_1)(\sin\theta_1)^{p-2} d\theta_1. \end{aligned}$$

According to Definition 4, the integral term of Z above can be expressed with $I_{p/2-1}(1)$ as:

$$\int_0^\pi \exp(\cos\theta_1)(\sin\theta_1)^{p-2} d\theta_1 = \frac{\Gamma\left(\frac{p-1}{2}\right)\Gamma\left(\frac{1}{2}\right)}{2^{1-p/2}} I_{p/2-1}(1).$$

Therefore, with the fact that $\Gamma\left(\frac{1}{2}\right) = \sqrt{\pi}$,

$$Z = \frac{2\pi^{\frac{p-1}{2}}}{\Gamma\left(\frac{p-1}{2}\right)} \frac{\Gamma\left(\frac{p-1}{2}\right)\Gamma\left(\frac{1}{2}\right)}{2^{1-p/2}} I_{p/2-1}(1) = (2\pi)^{p/2} I_{p/2-1}(1).$$

Plugging Z back to Equation (20), we finally arrive that

$$\lim_{|V|\to\infty} p(w_j \mid w_i) = \frac{1}{(2\pi)^{p/2} I_{p/2-1}(1)} \exp(\boldsymbol{v}_{w_j}^\top \boldsymbol{u}_{w_i}) = vMF_p(\boldsymbol{u}_{w_i}, 1).$$

Lemma 2. Let $\mathcal{X}$ be a set of n unit vectors drawn independently from the vMF distribution $vMF_p(\boldsymbol{\mu}, \kappa)$, *i.e.*,

$$\mathcal{X} = \{\boldsymbol{x}_i \in \mathbb{S}^{p-1} \mid \boldsymbol{x}_i \sim vMF_p(\boldsymbol{\mu}, \kappa), 1 \le i \le n\},$$

The maximum likelihood estimate for parameter $\boldsymbol{\mu}$ is given by the normalized sum of the n vectors, *i.e.*,

$$\hat{\boldsymbol{\mu}} = \frac{\sum_{i=1}^{n} \boldsymbol{x}_i}{\|\sum_{i=1}^{n} \boldsymbol{x}_i\|}.$$

PROOF. The likelihood of $\mathcal{X}$ is

$$P(\mathcal{X} \mid \boldsymbol{\mu}, \kappa) = \prod_{i=1}^{n} vMF_p(\boldsymbol{\mu}, \kappa) = \prod_{i=1}^{n} c_p(\kappa) \exp(\kappa \boldsymbol{\mu}^\top \boldsymbol{x}_i).$$

The log-likelihood is

$$\log P(\mathcal{X} \mid \boldsymbol{\mu}, \kappa) = n \log c_p(\kappa) + \kappa \boldsymbol{\mu}^\top \boldsymbol{s},$$

where $\boldsymbol{s} = \sum_{i=1}^{n} \boldsymbol{x}_i$.

Since $\|\boldsymbol{\mu}\| = 1$, we introduce a Lagrange multiplier η to account for the constraint and maximize the Lagrangian objective function below:

$$\mathcal{L}(\boldsymbol{\mu}, \kappa, \eta; \mathcal{X}) = n \log c_p(\kappa) + \kappa \boldsymbol{\mu}^\top \boldsymbol{s} + \eta(1 - \boldsymbol{\mu}^\top \boldsymbol{\mu}).$$

Then we compute the partial derivative of $\mathcal{L}(\boldsymbol{\mu}, \kappa, \eta; \mathcal{X})$ with regard to $\boldsymbol{\mu}$:

$$\frac{\partial \mathcal{L}(\boldsymbol{\mu}, \kappa, \eta; \mathcal{X})}{\partial \boldsymbol{\mu}} = \kappa \boldsymbol{s} - 2\eta \boldsymbol{\mu}.$$

After setting the partial derivative to be zero, we obtain

$$\hat{\boldsymbol{\mu}} = \frac{\hat{\kappa}}{2\hat{\eta}} \boldsymbol{s}. \tag{21}$$

Plugging Equation (21) into the constraint $\boldsymbol{\mu}^\top \boldsymbol{\mu} = 1$, we have

$$\hat{\eta} = \frac{\hat{\kappa}}{2} \|\boldsymbol{s}\|. \tag{22}$$

Substituting Equation (22) into Equation (21), we finally arrive at the maximum likelihood estimation of the mean direction:

$$\hat{\boldsymbol{\mu}} = \frac{\boldsymbol{s}}{\|\boldsymbol{s}\|} = \frac{\sum_{i=1}^{n} \boldsymbol{x}_i}{\|\sum_{i=1}^{n} \boldsymbol{x}_i\|}.$$

REFERENCES

Sra, S. (2007). *Matrix nearness problems in data mining*. Ph.D. thesis
